# Spatial Calibration of Weigh-In-Motion Systems—Evaluation of Metrological Properties

**DOI:** 10.3390/s26133978

**Published:** 2026-06-23

**Authors:** Janusz Gajda, Ryszard Sroka, Piotr Burnos, Mateusz Daniol

**Affiliations:** Department of Measurement and Electronics, AGH University of Krakow, Al. A. Mickiewicza 30, 30-059 Kraków, Poland; jgajda@agh.edu.pl (J.G.); burnos@agh.edu.pl (P.B.); daniol@agh.edu.pl (M.D.)

**Keywords:** dynamic weighing of road vehicles, weigh-in-motion (WIM) systems, system calibration, spatial calibration, WIM system accuracy

## Abstract

This article presents a method for calibration of dynamic vehicle weighing systems (WIM—Weigh-In-Motion) involving the calibration of all WIM stations operating within a given road network segment as a single process. A key assumption of the method is the presence of at least one scale with significantly higher accuracy than the calibrated systems in this part of road network. This reference scale function may be played by a static scale, slow-pass scale (LS-WIM—Low-Speed WIM) for measurement of vehicle axle load or by a selected WIM system with heightened accuracy. Both the reference scale and all systems undergoing calibration must be equipped with a system for the automatic recognition of vehicle registration number plates. The reference scale makes it possible to determine axle load values considered as benchmark values. Then, for each vehicle weighed on the reference scale and subsequently on any WIM system operating within the analysed area, the relative difference between the reference result and the WIM system measurement is calculated with respect to the reference value. This difference forms the basis for the operation of the algorithm estimating the coefficients of the static characteristic of the calibrated WIM system (so-called calibration coefficients), which are then used to determine corrected weighing results. The estimation of the coefficients is updated after each identified vehicle that has previously been weighed on the reference scale is considered. The article presents both the results of simulations and experimental studies concerning the proposed spatial method of calibration. The results obtained allow for an assessment of the effectiveness of the proposed solution. As can be seen from the analyses conducted, this method leads to a significant reduction in systematic error of vehicle weight measurement. Unfortunately, it does not eliminate random errors. The spatial calibration approach described in this paper has certain limitations. The main ones include the impact of ANPR system errors on calibration effectiveness, cases where a vehicle is unloaded or loaded between WIM stations, and the propagation of systematic errors from the reference systems to the other WIM systems. A significant advantage of the proposed spatial calibration method is that it can operate effectively using weighing data from a single reference WIM system and does not require heavy traffic volumes.

## 1. Introduction

The maintenance of road infrastructure and safety as well as the upholding of principles of fair competition in transport require vehicle weight checks to be conducted. This task can be performed both by certified inspectors and by automated systems, for example, with the use of dynamic vehicle weighing systems. WIM systems used for this purpose are called administrative WIM systems [1,2]. The widespread use of such systems requires the development of effective and efficient methods for their legalisation and subsequently periodic calibration as well as regularly performed verification of system operation quality.

The basic idea of the methods used for the metrological verification of WIM systems involves multiple passes through a calibrated weighing station of vehicles whose total mass (GVW—gross vehicle weight) and static axle load values were previously determined on reference scales, such as platform scales and static or low-speed (LS-WIM) scales. In practice, both the pre-weighed vehicle method [3,4] and the method based on vehicles that have previously passed through a WIM system and only then are weighed on a reference scale are used [5]. Other methods of calibration and quality verification of WIM systems are also proposed, such as continuous calibration [6,7] or static calibration [8,9].

Based on the obtained results, weighing errors are calculated as the difference between the measurement from the WIM system and the reference values. The GVW error is determined, as is the error in load on individual axles. The number and parameters of test vehicles as well as the metrological requirements for reference scales are defined in the relevant normative documents, for example, [10]. Both methods were described in detail in ASTM E1318-02 and in later works [11,12,13,14,15,16].

Nevertheless, both presented calibration methods are time-consuming and costly and require significant organisational effort. To limit the uncertainty of the calibration, it is necessary to perform at least a dozen or so test passes. For this reason, there has been an intensive search for new, more effective methods that would make it possible to shorten the calibration process while maintaining the required accuracy. Among the more interesting solutions are the conceptions of autocalibration described in, among others, [17,18,19,20,21].

At the same time, work is ongoing on the simplification of the process of weighing reference vehicles used in calibration itself. One proposal is to replace weighing on a full set of scales (platform scale for the measurement of GVW and static scales for measurement of axle load) with weighing solely on a low-speed scale (LS-WIM). In [4], it was shown that this solution considerably simplifies the procedure while maintaining a comparable accuracy.

This article presents the results of a study on a method for calibration of WIM systems known as the spatial method. The essence of this conception is the simultaneous calibration of all WIM systems located on a given part of the road network. A condition for the application of this method is the presence of at least one scale characterised by clearly higher accuracy than the other systems; this can be a static scale, an LS-WIM scale or an accurate WIM system used as a reference. Both the reference scale and the calibrated systems must be equipped with a system for recognition of vehicle registration number plates.

The primary method for maintaining high accuracy of the reference WIM system is sufficiently frequent calibration using the pre-weighed vehicle method. This approach is costly and time-consuming; however, methods based on statistical analysis of weighing results from such a system, as well as on trend observation and anomaly detection in the dataset, are known in the literature. Such analysis enables forecasting when suspected verification of the WIM system is required [22].

High accuracy of the reference WIM system can additionally be achieved through the selection of appropriate load sensor technology, increasing the number of sensors installed at the WIM site, proper selection of the system location, and installation in a high-quality pavement.

For every vehicle that is weighed on a reference scale, and which next has passed through any WIM system in the analysed area, the relative difference between both measurements is calculated regarding the reference value. This difference comprises the data for an estimation algorithm of the coefficient of the slope and zero offset of the static characteristics of a given WIM system. These parameters are updated each time a reference vehicle passes through the system. The need to take zero offset error into account has been demonstrated by previous studies [23].

The ideas for the method described here were originally presented in [24]. In the former, weighing errors were defined as the percentage difference between the results obtained in the WIM system and the static load of individual axles or axle groups. The calibration coefficients were determined so that the mean axle weighing error at the calibrated WIM station is equal to zero. It is worth pointing out here that in the model of static characteristics applied at that time, a simplification was adopted involving omitting the zero offset—and as a result only the parameter describing the slope of the characteristic was considered.

Methods known as network-assisted calibration and calibration transfer are based on a similar calibration concept. Network-assisted calibration relies on statistical analysis of traffic data obtained from measurements collected by multiple WIM stations and involving many different vehicles. The system must be capable of identifying that a particular vehicle has passed through several different WIM sites. Vehicle identification may be based on data such as ANPR (Automatic Number Plate Recognition) camera records, axle configuration, measured vehicle length, vehicle class, and the time of passage through a given WIM system [6,25,26].

From the large number of weighing results, those corresponding to heavy vehicles that regularly travel along a specific section of the road network are selected. Preference is given to vehicles exhibiting low variability in payload. Vehicles belonging to the transport fleet of a selected company and operating on fixed routes may also be used. Based on the measurement results obtained from these carefully selected vehicles, calibration factors, offset values, and temperature correction coefficients are determined for individual WIM systems.

The calibration transfer method is implemented within a network of sensors or measurement systems. It is based on the sequential transfer of calibration coefficients from one system to another, starting with the most accurate reference system [27]. A significant difference between the proposed spatial calibration method and the network-assisted calibration and calibration transfer approaches is that the former does not require a highly extensive network of WIM systems to operate effectively. Nevertheless, its application is more justified in areas where a considerable number of WIM systems are deployed. Furthermore, instead of transferring calibration coefficients sequentially from one system to the next, the proposed method enables calibration parameters to be transferred directly from a single reference WIM system to multiple WIM systems.

In this paper, results are presented of an assessment of the accuracy of a WIM system calibrated using a method in which the reference scale may be both a LS-WIM station or another WIM system with a known, high level of accuracy located in the same area of the road network. This latter approach is particularly interesting as it simplifies to a substantial degree the calibration process in organisational terms and can be implemented as an automated procedure. The algorithm developed for such a system indicates the current values of both coefficients of the static characteristics of the WIM system.

The presented calibration method does not reduce the random errors contained in the weighing results. There are several causes of random errors occurring during vehicle weighing in WIM systems. The primary cause is variations in suspended mass and wheel bounce induced by pavement unevenness. Additionally, changes in the speed of the weighed vehicle while passing through the WIM site, random selection of the vehicle’s trajectory through the WIM site, and electromagnetic interference affecting the data logger should also be mentioned.

The paper is organised as follows. In Section 2, the results of simulation studies conducted using the stream of vehicles method are presented. Section 3 is dedicated to experimental studies of the presented calibration method—the experimental station and the results of calibration performed at two WIM stations equipped with different load sensor technologies are described. The experiments were conducted both for the case in which the reference system is assumed to be a WIM system and in which this role is played by an LS-WIM scale. Section 4 summarises the results of the study and the main conclusions.

## 2. Simulation Studies

The aim of the simulation studies was an initial assessment of the effectiveness of the proposed spatial calibration method. Additionally, the simulations allowed for an analysis of the impact of parameters of the calibration process whose impact is difficult to assess using experimental methods. An example of such a parameter is the distance between the reference WIM system and the calibrated system.

### 2.1. Methodology

The effectiveness of the calibration process was assessed by comparing the accuracy of the WIM system before calibration and after calibration was performed. As a measure of accuracy, the reliability characteristics were used (1) along with the error δ_0.95_ [23].

*Pr*(|*x*|) = 1 − *Φ*(|*x*|) (1)

where |*x*| is the absolute value of the relative measurement error, and *Φ*(·) is the estimate of the cumulative distribution function of variable |*x*|.

The error δ_0.95_ is defined as a value of the relative weighing error whose probability of being exceeded is 0.05; that is, for the reliability characteristics (1), the following condition is met: Pr(δ_0.95_) = 0.05.

To conduct the simulation studies of the spatial calibration method for WIM systems, a simplified model was developed for two systems: a reference WIM system and the calibrated system. The metrological properties of both systems are described using the slope of the static characteristics (*slope*), the zero offset of these characteristics (*offset*), and the relative standard deviation (*sigma*) of the additive random component with a normal distribution and an expected value of zero, present in the weighing results.

Models of the vehicles were also developed, which were weighed in both WIM systems in the simulations. The vehicles were divided into two classes. The first comprised light vehicles with a gross vehicle weight in the range 1200–11,200 kg. The second comprised heavy vehicles whose GVW was 18,000–42,000 kg.

For both classes, average fuel consumption was assumed, i.e., 9 L/100 km for light vehicles and 25 L/100 km for heavy vehicles. Taking this parameter into account enabled an analysis of the impact of distance (*distance*) between the reference and calibrated systems on the effectiveness of the calibration process.

In the presented simulation experiment at the stage of system calibration, it was assumed that there would be a determined number of vehicles weighed on both the reference WIM system and the system undergoing calibration. In total, this process lasted four days (96 h), and the vehicles passed through the weighing station at a defined frequency *f_veh_*[1/24 h]. The number of vehicles considered in a single simulation was therefore directly determined by the value of this frequency. For example, for *f_veh_* = 10, 40 passes of calibrating vehicles were recorded.

The vehicle class (one of the two previously mentioned) and its gross weight (even distribution in defined variable ranges) were chosen randomly, in line with the models assumed for both vehicle classes.

System calibration involved the ongoing correction of its metrological properties by determining updated *slope_cor* and *offset_cor* values. These were calculated using the Recursive Least-Squares Estimator (RLS) [28]. The parameters of the calibrated WIM system were updated upon obtaining each new resulted of simulated calibration weighing.

The parameters of the assumed simulation models of both WIM systems and vehicle streams are shown in Table 1.

The values of the parameters *slope_cor* and *offset_cor* obtained during calibration were subsequently used to correct the results of current weighing in the calibrated WIM system. For each set of parameter values from Table 1, the simulation experiment was repeated 1000 times to obtain a sample with an appropriately large number of results allowing for a correct estimation of the assumed measures of accuracy, namely the reliability characteristics and the error of δ_0.95_.

Weighing errors occurring in WIM systems are the result of three key parameters that describe their metrological properties: static characteristics slope (*slope*), the zero offset of these characteristics (*offset*) and the level of random components present in the results (*sigma*). Their nominal values are, respectively, *slope* = 1.0, *offset* = 0.0 and *sigma* = 0.0. Every deviation from these values leads to the occurrence of weighing errors, both systematic and random.

To assess the effectiveness of the proposed spatial calibration method, it is essential to determine to what extent this calibration can correct the deviations of these parameters from their nominal values.

### 2.2. Simulation Results

The impact of distance (*distance*) between the reference and calibrated WIM systems was analysed, with the parameters of the reference system understood to be as follows: *slope_ref* = 1.0, *offset_ref* = 0.0 and *sigma_ref* = 0.0. For the calibrated system, in turn, the following values were assumed, *slope_cal* = 1.1, *offset_cal* = 0.0 and *sigma_cal* = 0.01, which is equivalent to a relative error of the slope of its static characteristics equal to 0.1. Additionally, the following parameters of the calibration process were determined: *f_veh_* = 100 and *distance* = *var*.

**Figure 1:**
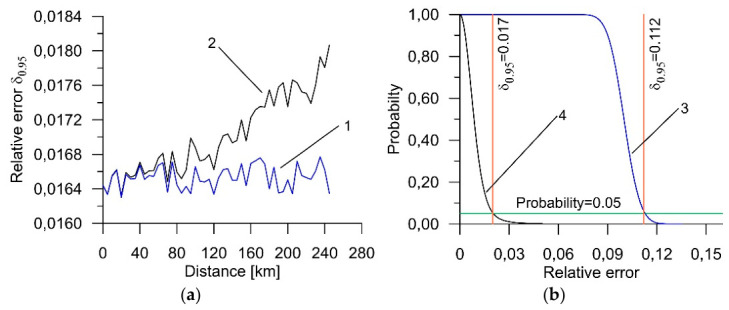
Impact of the distance between the reference WIM system and the calibrated system on the error δ_0.95_ of the calibrated system. (**a**) Relative error resulting from the distance between the systems: 1—relative error determined between the weighing results of the calibrated and reference systems, 2—error of the calibrated system relative to the actual vehicle weight. (**b**) Reliability characteristics of the calibrated system: 3—before calibration, 4—after calibration for the *distance* = 0.0 km.

In Figure 1a is an illustration of the impact of the *distance* between both WIM systems on the value of the error δ_0.95_ for the calibrated system. This error was determined regarding the indications of the reference system (curve 1) and the actual GVW of the vehicle (curve 2). Due to fuel consumption, the gross weight of the vehicle changes when passing through each of the systems, impacting the results obtained.

The reliability characteristics presented in Figure 1b allow the accuracy of the calibrated system to be compared before and after the performance of the calibration procedure.

From the characteristics shown in Figure 1a, it can be seen that after the performance of the correction of the static characteristics of the calibrated WIM system, its error δ_0.95_ does not exceed a value of 0.020 and is only slightly dependent on the distance between both systems. Before the performance of the calibration, due to a change in the slope of the static characteristics regarding the nominal value and random variability in the weighing results, the error δ_0.95_ achieved a value of 0.112. This conclusion is confirmed in the reliability characteristics presented in Figure 1b.

A key condition for the effective application of the spatial calibration method is to ensure that a sufficiently large number of vehicles is weighed both in the reference system and in the calibrated system. The probability of this is dependent on many factors, i.e., the distance between the systems, the number of available reference systems in the vicinity of the calibrated systems, the density of the road network and the intensity of traffic.

In the simulations presented here, the impact of the frequency *f_veh_* with which the vehicle is weighed successively in both WIM systems was analysed. With the assumption of a stable duration for the experiment (96 h, i.e., four days), this frequency is the deciding factor in the number of vehicles that are weighed twice.

**Figure 2:**
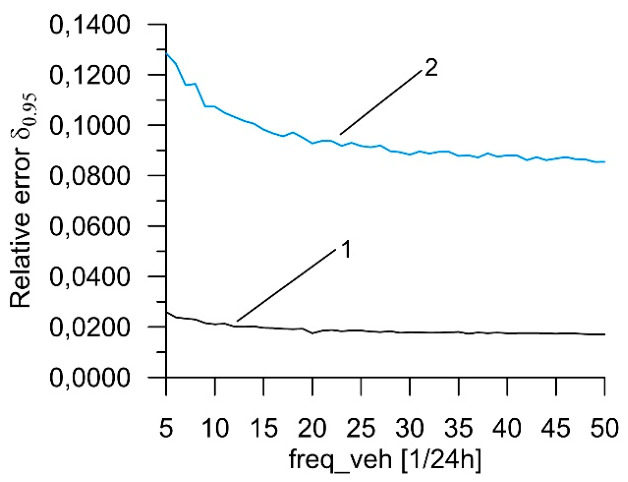
Impact of the frequency of vehicle weighing in both WIM systems on the error δ_0.95_ of the calibrated system: 1—error of the calibrated system relative to the actual vehicle weight, for *sigma_cal* = 0.01, 2—error of the calibrated system relative to the actual vehicle weight, for *sigma_cal* = 0.05.

In the simulation experiment, which aimed to determine the impact of the frequency *f_veh_* of weighing the vehicles in both systems, the following values of the reference WIM system were assumed: *slope_ref* = 1.0, *offset_ref* = 0.0, *sigma_ref* = 0.0; and for the calibrated WIM system: *slope_cal* = 1.1, *offset_cal* = 0.0, *sigma_cal* = 0.01 and *sigma_cal* = 0.05. The parameters of the calibration process were established as *f_veh_* = *var*, *distance* = 0.0 km. the obtained results are shown in Figure 2.

The frequency of weighing the vehicles in both WIM systems does not exert a significant impact on the effectiveness of the calibration process. Even weighing a mere 10 vehicles over a day in both systems was sufficient to limit the error of the calibrated WIM system to a level resulting strictly from the random variability of weighing results in the system. This is a crucial observation, as in practice only a small number of the vehicles passing through the reference WIM system also pass through the system undergoing calibration.

**Figure 3:**
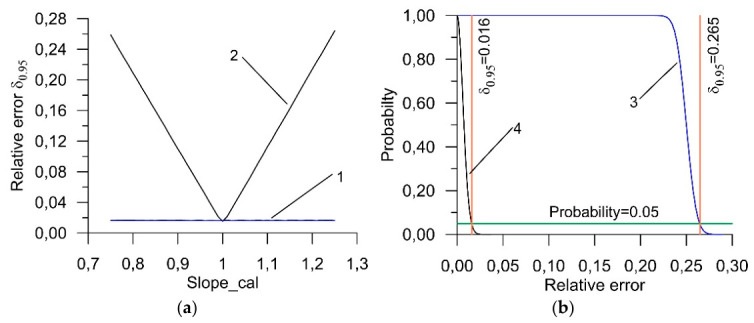
Impact of the parameter *slope_cal* of the static characteristics of the calibrated WIM system on the effectiveness of the spatial calibration process as assessed by the error δ_0.95_. (**a**) Relative error resulting from the *slope_cal* parameter: 1—error of the calibrated system after calibration, 2—error of the calibrated system when calibration is not performed. (**b**) Reliability characteristics of the calibrated WIM system before calibration: 3—*slope_cal* = 1.25, 4—*slope_cal* = 1.0.

The effectiveness of the correction of the slope of the static characteristics of the calibrated WIM system, and as a result the effectiveness of the spatial calibration, can be assessed with the aid of the characteristics shown in Figure 3. During the simulation experiment conducted, the following values for parameters were assumed. For the reference WIM system: *slope_ref* = 1.0, *offset_ref* = 0.0, *sigma_ref* = 0.0. For the calibrated WIM system: *slope_cal* = *var*, *offset_cal* = 0.0, *sigma_cal* = 0.01. The parameters of the calibration process were established as *f_veh_* = 100, *distance* = 0.0.

The reliability characteristics shown in Figure 3b reflect two points in the characteristics 2 from Figure 3a whose *x*-axis values are equal to the set values of the slope of the static characteristics *slope_cal*, for curves 3 and 4, respectively.

The slope of the static characteristics of the calibrated WIM system (*slope_cal*) causes a relative systematic error equal to the difference between the actual value of the slope and the ideal (nominal) value, that is *slope_cal* = 1.0. This dependency is shown by curve 2 in Figure 3a. The application of the spatial calibration algorithm allows for a reduction in the total weighing error to a level determined solely by the random variability of weighing results in the calibrated system (curve 1).

In the simulation experiment, which aimed to determine the effectiveness of calibration in compensation for the impact of the parameter *offset_cal*, the following values of the parameters were assumed. For the reference WIM system: *slope_ref* = 1.0, *offset_ref* = 0.0, *sigma_ref* = 0.0. For the calibrated WIM system: *slope_cal* = 1.1 and *slope_cal* = 1.0, *offset_cal* = var, *sigma_cal* = 0.01. The parameters of the calibration process were established as *f_veh_* = 100, *distance* = 0.0.

**Figure 4:**
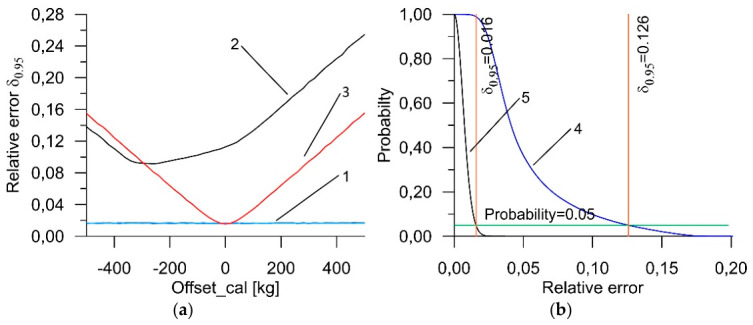
Impact of the zero offset of the static characteristics of the calibrated WIM system (*offset_cal*) on the effectiveness of the spatial calibration process as assessed by the error δ_0.95_. (**a**) Relative error resulting from the *offset_cal* parameter: 1—error of the calibrated system after calibration, 2—error of the calibrated system before calibration when the parameter *slope_cal* = 1.1, 3—error of the calibrated system before calibration when the parameter *slope_cal* = 1.0. (**b**) Reliability characteristics of the calibrated WIM system before calibration: 4—*slope_cal* = 1.0, *offset_cal* = 400 kg, 5—*slope_cal* = 1.0, *offset_cal* = 0.0.

Conclusions regarding the effectiveness of calibration can be drawn from the characteristics shown in Figure 4.

The zero offset of the static characteristics of the calibrated WIM system (*offset_cal*) causes a substantial increase in the relative vehicle weighing error (curves 2 and 3 in Figure 4a and curve 4 in Figure 4b). It should be remembered that a zero offset with a value of, for example, 400 kg will result in various values of relative weighing error depending on the gross weight (GVW) of the vehicle.

The proposed spatial calibration procedure, in this case as well, can reduce the value of the weighing error δ_0.95_ to a level resulting strictly from the random component included in the weighing results.

There are several reasons for the random errors occurring in the results of dynamic weighing of vehicles in WIM systems. The main ones, however, are vertical movements of the vehicle as it is passing over the weighing station and the bouncing of individual axles as a response to unevenness of the pavement which the vehicles are travelling on. In the simulations, this effect was modelled with an additive random component with a normal distribution, zero expected value and relative standard deviation *sigma*, regarding the value of the quantity measured, i.e., GVW. The value of the error δ_0.95_ is an effect of the combined occurrence of systematic errors resulting from the parameters of the static characteristics of the WIM system and random errors.

**Figure 5:**
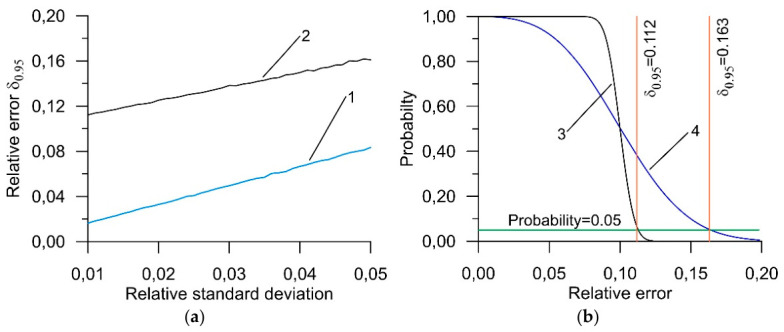
Impact of the relative standard deviation *sigma_cal* of the additive random component included in the weighing results of the calibrated WIM system on the effectiveness of the spatial calibration process as assessed by the error δ_0.95_. (**a**) Relative error resulting from the *sigma_cal* parameter: 1—error of the calibrated system after calibration, 2—error of the calibrated system before calibration. (**b**) Reliability characteristics of the calibrated WIM system before calibration: 3—*slope_cal* = 1.1, *offset_cal* = 0.0, *sigma_cal* = 0.01, 4—*slope_cal* = 1.1, *offset_cal* = 0.0, *sigma_cal* = 0.05.

An assessment of the effectiveness in limiting the impact of random error on the accuracy of weighing using the spatial calibration method for WIM systems can be drawn from the characteristics shown in Figure 5. These were determined with the assumption of the following values of the reference WIM system: *slope_ref* = 1.0, *offset_ref* = 0.0, *sigma_ref* = 0.0. The parameters of the calibrated WIM system were *slope_cal* = 1.1, *offset_cal* = 0.0, *sigma_cal* = var, and the parameters of the calibration process were *f_veh_* = 100, *distance* = 0.0.

The additive random interference present in the weighing results in the calibrated WIM system reduces the effectiveness of the applied spatial calibration procedure. This method, however, does not make it possible to eliminate the impact of this interference. On the other hand, despite the occurrence of the random component, calibration enables the effective elimination of the error of the slope of the static characteristics of the system.

**Figure 6:**
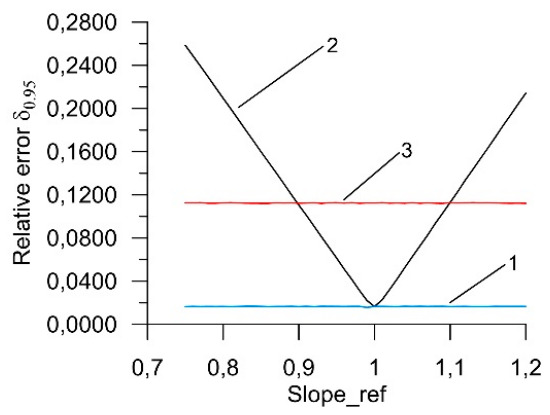
Effect of the *slope_ref* parameter of the static characteristic of the reference WIM system on the effectiveness of spatial calibration: 1—relative error δ_0.95_ calculated from differences between the weighing results of the reference and calibrated systems; 2—error of the calibrated system relative to the true vehicle weight; 3—error of the calibrated system before calibration.

**Figure 7:**
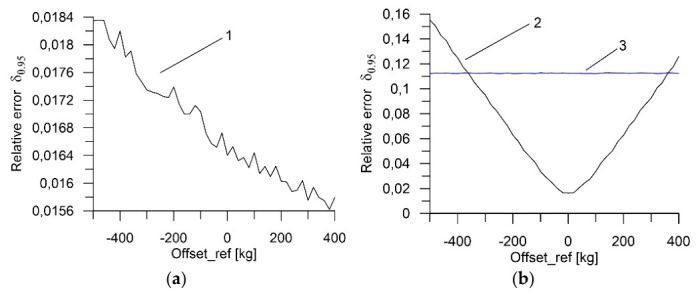
Effect of the zero-offset parameter, (*offset_ref*), of the static characteristic of the reference WIM system on the effectiveness of spatial calibration. (**a**) Relative error δ_0.95_ calculated from the differences between the weighing results of the reference and calibrated systems (curve 1). (**b**) Relative error resulting from the *offset_ref* parameter: 2—error of the calibrated system relative to the true vehicle weight; 3—error of the calibrated system before calibration.

**Figure 8:**
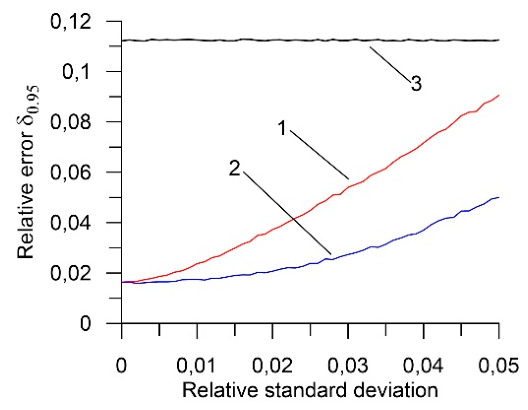
Effect of the relative standard deviation (*sigma_ref*) of the additive random error component in the weighing results of the reference WIM system on the effectiveness of the spatial calibration process: 1—relative error δ_0.95_ determined from the differences between the weighing results obtained from the reference and calibrated systems; 2—error of the calibrated system with respect to the true vehicle weight; 3—error of the calibrated system before calibration.

Undoubtedly, the accuracy of the calibration process is also influenced by the quality of the reference system. To assess how errors of the reference system affect the accuracy of the calibrated system, the characteristics shown in Figure 6, Figure 7 and Figure 8 were determined.

Figure 6 illustrates the effect of the slope parameter (*slope_ref*) of the static characteristic of the reference WIM system on the effectiveness of the spatial calibration process. The effectiveness is evaluated using the relative error δ_0.95_. During the study, the following parameter values were assumed for both systems: *slope_ref* = var, *offset_ref* = 0.0, *sigma_ref* = 0.0, *slope_cal* = 1.1, *offset_cal* = 0.0, *sigma_cal* = 0.01.

The characteristics presented in Figure 6 indicate that the parameters of the calibrated system tend to follow those of the reference system. As a result, the accuracy of the calibrated system deteriorates as the value of *slope_ref* deviates from its optimal value. Consequently, calibration performed using a reference system with an improperly adjusted slope parameter transfers this error to the calibrated system, reducing the effectiveness of the spatial calibration process.

A similar conclusion can be drawn from the characteristics presented in Figure 7, which illustrate the effect of the zero-offset error of the static characteristic of the reference WIM system. During the study, the following parameter values were assumed for the two systems: *slope_ref* = 1.0, *offset_ref* = var, *sigma_ref* = 0.0, *slope_cal* = 1.1, *offset_cal* = 0.0, *sigma_cal* = 0.01.

The results demonstrate that the offset error of the reference system is also transferred to the calibrated system. Consequently, as the value of *offset_ref* deviates from zero, the accuracy of the calibrated system decreases, confirming that the effectiveness of the spatial calibration procedure strongly depends on the metrological quality of the reference WIM system.

The influence of random errors in the reference WIM system on the accuracy of the calibrated WIM system is presented in Figure 8. The following parameter values were assumed for the reference and calibrated systems: *slope_ref* = 1.0, *offset_ref* = 0.0, *sigma_ref* = var, *slope_cal* = 1.1, *offset_cal* = 0, *sigma_cal* = 0.01.

The random component present in the weighing results of the reference system degrades the accuracy of the calibrated system. However, it should be emphasized that this adverse effect is less pronounced than the impact of the same random component on the accuracy of the reference system itself.

The results presented in Figure 6, Figure 7 and Figure 8 demonstrate that errors present in the reference system are propagated to the calibrated system, leading to a reduction in its measurement accuracy. Consequently, the accuracy characteristics of both WIM systems converge, meaning that the calibrated system cannot be expected to exhibit a higher weighing accuracy than the reference WIM system upon which the calibration process is based.

## 3. Experimental Studies

**Figure 9:**
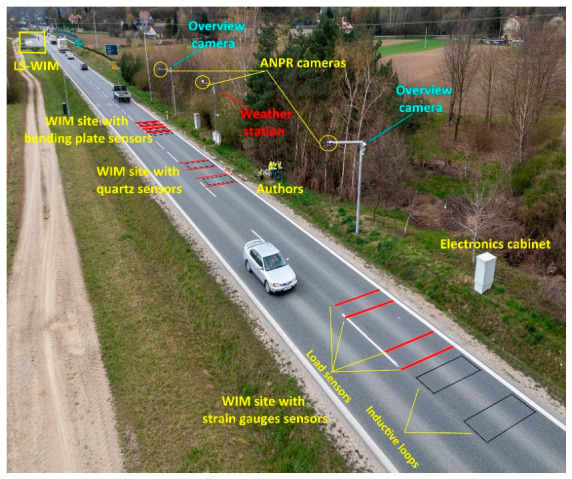
Sample view of the WIM experimental station.

The experimental studies were conducted at a field research station located along provincial route no. 975 in southern Poland [29]. A sample view of the station is shown in Figure 9. The station features three WIM systems, each of them equipped with four lines of load sensors constructed with different technologies. These are strain gauge bending plate sensors, quartz sensors and strip strain gauge sensors.

Due to the various operating lifetimes of the individual systems and the varied technologies and construction methods used, the WIM systems are characterised by different levels of accuracy. In the immediate vicinity of the WIM stations, there is a low-speed scale (LS-WIM), making it possible to measure the static load of individual axles of the vehicle. In compliance with its current legalisation certification, the scale features an accuracy class of D2.

### 3.1. Methodology

The measurement experiments involved the recording of weighing results of heavy vehicles passing through all three WIM stations. Additionally, these vehicles were weighed on an LS-WIM scale. The weighed vehicles differed in their construction—they included a two-axle rigid-body vehicle, a three-axle vehicle and a five-axle vehicle comprising a two-axle tractor and a three-axle trailer.

Measurements obtained at a WIM station equipped with strip strain gauges sensors and weighing results from the LS-WIM scale were used as reference values. The quality of the reference WIM system was assessed by comparing it with the results obtained from the LS-WIM scale. The WIM systems equipped with bending plate sensors and quartz sensors, on the other hand, were systems calibrated using the spatial method.

The experiments were performed three times: in October 2024, in January 2025 and in April 2025. During each of these, each of the three vehicles passed through the WIM stations a dozen (15–20) times. This allowed for a total of 45 to 60 weighings of vehicles belonging to the three classes. The obtained measurement data allowed for the determination of reliability characteristics of the calibrated WIM systems, both before and after calibration.

### 3.2. Results of a Study of a WIM System Equipped with Bending Plate Sensors

**Figure 10:**
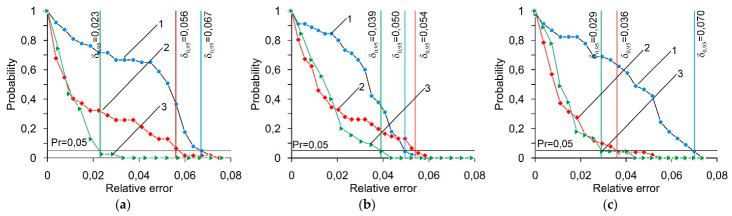
Reliability characteristics of the reference and calibrated WIM system (bending plate). (**a**) In October 2024. (**b**) In January 2025. (**c**) In April 2025. 1—calibrated WIM before calibration, 2—calibrated WIM after calibration, 3—reference WIM system.

Figure 10 shows the reliability characteristics of a WIM system obtained in subsequent measurement experiments. Calibration performed using the spatial method allowed for a reduction of error δ_0.95_. The best results of the applied method were obtained in January 2025.

In analysing the results shown in Figure 10, attention should be paid not only to the error δ_0.95_ but also to the shape of the reliability characteristics, which depends on the probability of the occurrence of an error of a determined value. The lowering of the course of the characteristics obtained because of calibration indicates a reduction in the probability of the occurrence of an error of a given value. On this basis, it can be stated that the applied method of spatial calibration considerably improved the accuracy of weighing.

Table 2 presents the relative values of the standard deviation and RMSE (Root Mean Square Error) of the weighing results obtained during the three experimental campaigns. The results presented confirm the conclusions drawn from the simulation studies, indicating that it is not possible to reduce the random variability of the weighing results while simultaneously achieving a significant reduction in RMSE.

**Figure 11:**
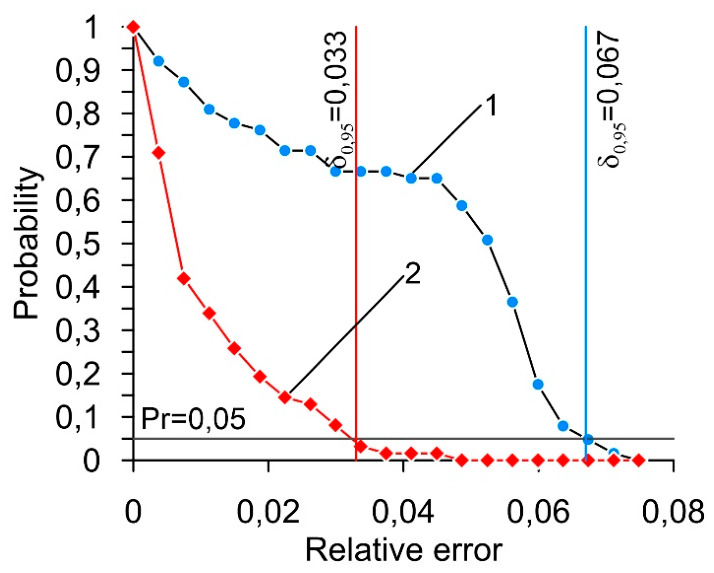
Reliability characteristics of the calibrated WIM system (bending plate) in October 2024 when an LS-WIM scale was used as a reference. 1—before calibration, 2—after calibration.

For comparison, an analogous set of reliability characteristics was determined for the case in which the reference system was an LS-WIM scale. This procedure is compliant with applicable normalisation documents [10]. The relevant characteristics are presented in Figure 11.

In the case of calibration performed using the traditional method, i.e., using vehicles previously weighed on an LS-WIM scale, the error δ_0.95_ after calibration was 0.033, compared to the value of 0.067 before calibration. This result is comparable to results obtained using the proposed spatial calibration method, for example, in April 2025 (Figure 10).

It should, however, be noted that the weighing results obtained using the reference WIM system are characterised by significant random error when compared to the data from the LS-WIM scale. The effect of this error is clearly visible when juxtaposing the characteristics presented in Figure 10 with those in Figure 11. Despite this, the reduction in systematic error in the calibrated WIM system obtained thanks to spatial calibration is significant, as is well illustrated by the characteristics in Figure 10.

This result is, however, generally worse than the effects of calibration based on the results of weighing reference vehicles on a reference LS-WIM scale.

A *t*-test performed for the WIM system calibrated using data from the LS-WIM scale and the reference WIM system indicated that the differences between the weighing results obtained in both cases are due solely to their random variability (*p*-value = 0.997).

Nonetheless, an additional advantage of the spatial calibration method is the possibility of automating it, meaning that the operation of the calibration procedure can be continuous. The pre-weighed vehicles method requires that vehicles be rented, generating costs and occupying the time of LS-WIM scale staff, thus limiting the application of this method to experiments conducted at most once every few months.

### 3.3. Results of a Study of a WIM System Equipped with Linear Quartz Sensors

**Figure 12:**
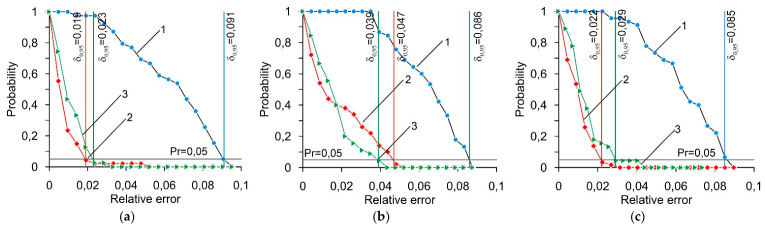
Reliability characteristics of the reference and calibrated WIM system (quartz sensors). (**a**) In October 2024. (**b**) In January 2025. (**c**) In April 2025. 1—calibrated WIM before calibration, 2—calibrated WIM after calibration, 3—reference WIM system.

Analogous characteristics were determined for a WIM system equipped with quartz load sensors, and the results are presented in Figure 12. In the case of a system like this, the advantages entailed by calibration using the spatial method are clearly greater, as can be seen from the lower level of random error present in the weighing results of the calibrated system. As was shown in the simulation studies, random error cannot be limited through system calibration. On the other hand, systematic error resulting from the deviation of coefficients of the static characteristics from nominal values can be considerably reduced using this method. Results obtained experimentally confirm this conclusion.

**Figure 13:**
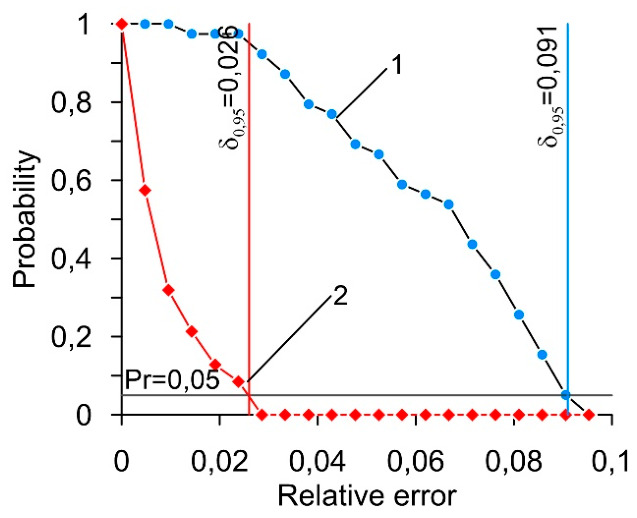
Reliability characteristics of the calibrated WIM system (quartz sensors) in October 2024 when an LS-WIM scale was used as a reference. 1—before calibration, 2—after calibration.

In the case of calibration performed using the traditional method, with the use of vehicles previously weighed on an LS-WIM scale, the error δ_0.95_ after calibration was 0.026, in contrast to 0.091 before calibration (Figure 13). This is a result that is slightly worse than that obtained using the spatial calibration method in October 2024 (Figure 12a). The uncertainty of the estimation of this error value must, however, be considered.

A *t*-test performed for the WIM system calibrated using data from the LS-WIM scale and the reference WIM system indicated that the differences between the weighing results obtained in both cases are due solely to their random variability (*p*-value = 0.910).

The results presented in Table 3 confirm the conclusions drawn earlier based on Table 2.

**Figure 14:**
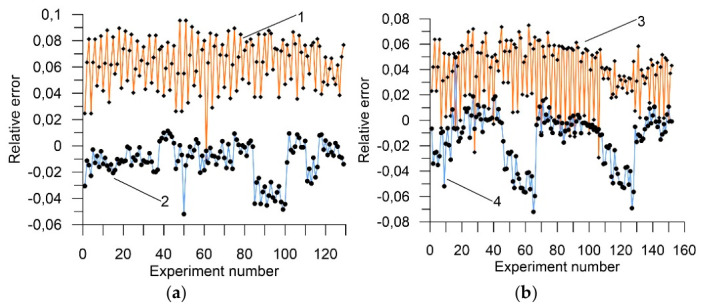
Relative weighing errors obtained for the two investigated WIM systems. (**a**) System with quartz sensors: 1—before calibration; 2—after calibration. (**b**) System with bending plate sensors: 3—before calibration; 4—after calibration.

Figure 14 presents the errors of both WIM systems before and after calibration. For the WIM system equipped with quartz sensors, the mean relative error was reduced by approximately a factor of six because of the calibration procedure, decreasing from an initial value of 0.063 to −0.011.

For the WIM system equipped with bending plate sensors, the mean relative error was reduced by more than a factor of two, from 0.036 to −0.014. The lower calibration effectiveness observed for this system can be attributed to the larger contribution of random errors relative to the systematic error component. This conclusion is further supported by the characteristics presented in Figure 10 and Figure 12, which indicate a higher level of random variability in the weighing results obtained from this system.

## 4. Conclusions

This paper presents the results of a study of a method of spatial calibration of WIM systems. The study involved simulations of the proposed method and six experiments at WIM stations in the field equipped with load sensors using two different technologies. The experiments were performed in varying environmental conditions, which changed along with the changing seasons.

The presented spatial calibration method for WIM systems allows for a significant reduction in the systematic error of weighing, although it does not eliminate random error. Among the advantages of this system is the fact that it does not require the use of additional infrastructure such as an LS-WIM scale, portable scales or platform scales located in the vicinity of the calibrated system. Other significant advantages of this calibration method are the possibility of using a variety of reference vehicles whose GVWs represent a broad range of values, thanks to which the entire measurement range of the calibrated WIM system is covered, and the possibility of conducting such calibration on an ongoing basis.

The spatial calibration method delivers slightly worse results than the traditional calibration method, i.e., with the use of vehicles previously weighed on an LS-WIM scale. These disparities, however, did not exceed 1.8% for the system with bending plate sensors and 1% for the system with quartz sensors. It is additionally possible to apply the spatial calibration method using reference vehicles previously weighed on an LS-WIM scale—this solution may increase the accuracy of the WIM system, but it requires the involvement of specialised personnel.

The WIM system with quartz sensors is characterised by a lower level of random error than the system with bending plate sensors. As a result, its reliability characteristics after spatial calibration are very similar to the characteristics of the reference system, as is the error value δ_0.95_.

The results presented, confirming the effectiveness of the proposed method, were obtained for a relatively small number of vehicles weighed both in a reference system and in the calibrated system. This is a further advantage of this system—its effectiveness is dependent only to a small degree on the number of available reference vehicles, as was also confirmed by the results of the simulations.

For the practical implementation of the spatial method of calibration, three conditions must be met:

- Within a limited area, there must be a reference WIM system whose accuracy is maintained using the traditional calibration methods, performed frequently enough to ensure a high level of accuracy; this role may be played by an LS-WIM scale;
- Vehicles weighed on the reference system must also appear with a non-zero probability at the calibrated WIM station;
- The calibrated system must be able to identify these vehicles and obtain their weighing results from the reference system.

The fulfilment of these conditions, with the currently available technologies and with a sufficiently dense network of WIM stations, should not pose serious difficulties.

The applied RLS algorithm is not particularly computationally demanding. Each iteration involves only basic addition and multiplication operations performed on scalar, vector, and matrix variables. However, since only two calibration coefficients are estimated during the calibration process, the vector variables contain only two elements, and the matrix has dimensions of 2 × 2.

To avoid errors caused by the loading or unloading of a vehicle that could occur between the reference WIM system and the WIM system being calibrated, it is necessary to define the maximum allowable time delay between the weighings of the same vehicle. The permissible delay should consider the distance between the two WIM sites, the applicable speed limit, traffic conditions, and the estimated time required for a potential loading or unloading operation for a given vehicle class.

Also, if the ANPR system malfunctions at any WIM station (reference or calibrated), the proposed spatial calibration method will not function fully correctly. The system will continue to operate using the existing current calibration coefficients, and no further updates to these coefficients will be made until synchronization between the systems is restored.

## Figures and Tables

**Table 1:** Indicators for the parameters of the vehicle weighing process model.

Parameter Name	Parameter Description
*slope_ref*	Slope of the static characteristics of the reference scale
*offset_ref*	Zero offset of the static characteristics of the reference scale
*sigma_ref*	Standard deviation of the additive random component included in the weighing results on the reference scale
*slope_cal*	Slope of the static characteristics of the calibrated WIM system
*offset_cal*	Zero offset of the static characteristics of the calibrated WIM system
*sigma_cal*	Standard deviation of the additive random component included in the weighing results in the calibrated WIM system
*f_veh_*[1/24 h]	Number of vehicles weighed first on the reference scale and subsequently on the calibrated WIM system within a single day
*distance* [km]	Distance between the reference scale and the calibrated WIM system

**Table 2:** Relative standard deviation and RMSE for weighing results on WIM site equipped with bending plate load sensors.

	Before Calibration		After Calibration	
	Standard Deviation	RMSE	Standard Deviation	RMSE
October 2024				
2-axle	0.0147	0.0144	0.0138	0.0238
3-axle	0.0085	0.0568	0.0079	0.0081
5-axle	0.0049	0.0570	0.0046	0.0061
January 2025				
2-axle	0.0149	0.0190	0.0145	0.0422
3-axle	0.0083	0.0334	0.0081	0.0096
5-axle	0.0083	0.0406	0.0080	0.0088
April 2025				
2-axle	0.0242	0.0270	0.0229	0.0271
3-axle	0.0110	0.0492	0.0104	0.0105
5-axle	0.0092	0.0582	0.0087	0.0099

**Table 3:** Relative standard deviation and RMSE for weighing results on WIM site equipped with quartz load sensors.

	Before Calibration		After Calibration	
	Standard Deviation	RMSE	Standard Deviation	RMSE
October 2024				
2-axle	0.0164	0.0382	0.0148	0.0199
3-axle	0.0076	0.0675	0.0069	0.0083
5-axle	0.0052	0.0843	0.0047	0.0048
January 2025				
2-axle	0.0079	0.0447	0.0074	0.0382
3-axle	0.0133	0.0707	0.0124	0.0141
5-axle	0.0067	0.0743	0.0063	0.0065
April 2025				
2-axle	0.0076	0.0427	0.0070	0.0160
3-axle	0.0058	0.0630	0.0054	0.0121
5-axle	0.0042	0.0811	0.0039	0.0051

## Data Availability

Inquiries for data can be directed to the corresponding author.
